# Changes of The Uterine Tissue in Rats with Polycystic Ovary Syndrome Induced by Estradiol Valerate

**DOI:** 10.22074/ijfs.2016.4794

**Published:** 2016-11-11

**Authors:** Ghadire Mirabolghasemi, Zahra Kamyab

**Affiliations:** Department of Animal Biology, Faculty of Biological Sciences, Kharazmi University, Tehran, Iran

**Keywords:** Uterus, Estradiol Valerate, Polycystic Ovary Syndrome, Mitosis, Rat

## Abstract

**Background:**

Polycystic ovary syndrome (PCOS) is one of the most common hormonal disorders that can lead to irregular menstrual cycles and hyperandrogenism. Reduced levels of progesterone and increased estrogen in these women can perpetually stimulate the endometrial tissue of the uterus. In this study, we assess the effect of PCOS induction by estradiol valerate (EV) in a rat model.

**Materials and Methods:**

In this experimental study, adult female Wistar rats that weighed approximately 200 g were divided into control, sham, and experimental groups (n=6 per group). The experimental group received subcutaneous injections of 2 mg EV for induction of PCOS. We confirmed the presence of PCOS in the experimental group rats. Rats from all groups were subsequently killed, after which their uteri were removed and fixed for histological and cytological analyses. The uterine tissue sections were stained with hematoxylin and eosin (H&E) and iron hematoxylin (iron-H). We examined epithelium height, thickness of the uterus wall, and frequency of the mitotic cells. The data were assessed at α=0.05.

**Results:**

Uterine tissue findings from the experimental group showed significant increases in the height of the uterus luminal epithelium, the thickness of the uterus wall, and the frequency of eosinophils in the endometrial stroma. We observed an increased frequency of mitotic cells in the experimental group in both luminal and glandular epithelia of the uterus. An increased rate of the glandular epithelium region was noticeable and significant.

**Conclusion:**

Induction of PCOS by EV could change the proliferation rate in the endo- metrial tissue of the uterus.

## Introduction

Polycystic ovary syndrome (PCOS) is a hormonal imbalance disorder (1, 2) that occurs in approximately 4-18% of reproductive-aged women (12 to 45 years) (3). PCOS is a metabolic and reproductive disorder with characteristic features that include hyperandrogenism, irregular menstrual cycles, insulin resistance, obesity, hirsutism, and acne (4). Anovulation that results from PCOS is the most common cause of infertility in women (5). Features of PCOS may manifest at any age and range from childhood (premature puberty), teenage (hirsutism, menstrual abnormalities), early adulthood and middle life (infertility, glucose intolerance), to later life (diabetes mellitus and cardiovascular diseases) (6).

Numerous evidences affirm the fact that endocrinologic and metabolic abnormalities in PCOS may have complex effects on endometrial tissue, thus contributing to infertility and endometrial disorders in women with this syndrome (7). Long-term PCOS increases the risk of hyperplasia, endometrial cancer (EC), and metabolic syndrome (8). Endometrial hyperplasia is a premalignant condition that usually heralds EC (9). It has been reported that women with PCOS and endometrial hyperplasia have a four times greater risk of developing EC than women without PCOS (10). Hyperplasia and uterine cancer have been observed in women with PCOS who received no treatment (11). 

The two main types of EC are estrogen-dependent type Ι and estrogen-independent type ΙΙ (12). It is widely believed that PCOS is one of the most impressive risk factors that promote type I EC (10, 13, 14). Prolonged exposure of the endometrium to estrogen, as a consequence of anovulation, is suggested to be the prime cause of this increased risk (15). Therefore, the hormonal imbalance associated with PCOS can alter endometrial tissue homeostasis and promote cell proliferation (16). In humans, continuous exposure of the endometrium to estrogen can lead to endometrial hyperplasia (17). Progesterone acts as a protective factor against estrogen-driven uterine growth and proliferation (18).

Steroid hormone levels regulate the cycle of cellular proliferation and apoptosis in the endometrial tissue. Therefore, a firm balance between these two processes would secure the normal function of the endometrium (19). Endocrine-metabolic situations associated with abnormalities in plasma hormone concentrations, as seen with PCOS, can affect the processes that occur in the endometrium, which includes cell proliferation, differentiation and response to biological stimuli (20). Estrogen is a hormone that affects the uterus. Strong activation of proliferative activity is the most important physiological effect of estrogen hormones in the uterus (21). Significant consequences of (particularly long-term) endometrial exposure to estrogen are morphogenetic alterations that include modified type of luminal and glandular epithelia, glandular shape, and the glandular to stromal ratio (22, 23).

Estradiol valerate (EV) is used to create PCOS by inducing hormone abnormalities (24). EV, which is introduced as a prodrug, is an ester derived from 17β-estradiol. EV is normally cleared in blood plasma and the liver into 17β-estradiol by esterase activity (25). The 17β-estradiol metabolizing procedure includes an array of reversible and non-reversible enzyme-mediated reactions (26). The metabolites 17β-estradiol and estron may predict the risk of breast (27) and other hormone-related cancers (28). Studies show that hormonal abnormalities attributed to EV can create a phenotype similar to PCOS (29). In this study we focus on tissue changes and proliferation activity of the uterus in a rat model of PCOS induced by EV.

## Materials and Methods

### Animals

The present experimental study used 18 adult female Wistar rats that weighed 200 ± 20 g. Animals were obtained from the Pharmacology Department of Tehran University and maintained in special cages under standard conditions of 22ºC, a 12-hour dark/light cycle, and free access standard chow and water. In order to conduct a comparative evaluation, we divided the rats into three groups of 6 animals per group: control (normal rats), experimental group or PCOS (rats that received EV), and sham (rats that received EV solvent). Before the induction, we confirmed the rats’ normal estrous cycles through daily vaginal smears over two weeks. Animals that had at least two normal estrus cycles were selected for PCOS induction.

The Ethics Committee of the Biological Sciences Faculty at Kharazmi University, Tehran, Iran approved this study. 

### Induction of polycystic ovary syndrome

We used EV to induce the polycystic condition. Each experimental rat received 2 mg of EV, dissolved in 0.2 ml sesame oil, through a single subcutaneous injection at the inguinal region. Rats in the sham group received an equal volume of sesame oil. Subsequently, vaginal smears of these rats were monitored for 60 days, until the time when abnormal estrus cycles and persistent vaginal cornification (PVC) occurred as a sign of the presence of ovarian cysts and early confirmation of PCOS induction (30). Rats in the sham group that received sesame oil showed no evidence of abnormalities in estrus cycles or vaginal smears. Hence, further experiments were concentrated mainly on control and PCOS rats.

### Histological and cytological studies 

On the 60^th^ day after the EV injection, rats from all groups were sacrificed and the uterine specimens were fixed in 10% formaldehyde. The tissue samples were dehydrated by graded series of ethanol, embedded in paraffin, then sectioned into 5-7 µm sections prior to microscopic analysis. 

### Histological evaluations of the uterus and determination of mitosis

As mentioned, the effect of estrogens on the uterus tissue is chiefly related to its strong invigorating impact on cell proliferation. Long-term exposure to estrogen leads to uterus endometrium overgrowth and hyperplasia. We used hematoxylin and eosin (H&E) in addition to iron hematoxylin (iron-H) staining to conduct in-depth assessments of histological changes, the occurrence of mitosis, and proliferating cells.

For histological evaluations, tissue sections were stained with H&E. We measured the height of the epithelial cells, uterus wall thickness, accumulation of uterine glands, and the number of eosinophil cells in the uterine stroma as visualized by a light microscope at ×100, ×400, and ×1000 magnifications. The longitudinal measurements were obtained by Microstructure Measurement software ver.1.04 (Scalor, Crop Toky, Japan). 

For iron-H staining, we stained the tissue sections with Heidenhain’s iron hematoxylin color. In this hematoxylin solution, iron salts are used both as an oxidizer and a mordant. This staining method can be used to demonstrate numerous structures, such as nuclear chromatin, according to the degree of differentiation (31). This staining method shows the presence of cells during the mitotic cycle. In order to measure the percentage ratio of proliferating cells to the total number of epithelial cells, we separately counted both the total and mitotic cell numbers in the uterus luminal and glandular epithelia in 10 microscopic fields of view for each tissue specimen at ×1000 magnification with a light microscope. Overall, we assessed 4359 cells.

### Statistical analysis

Comparative assessments of the aforementioned parameters are reported as mean ± SE. Assessment between PCOS and the control group was performed through one-way ANOVA (Tukey post hoctest) by SPSS Statistics software ver. 20.0 (IBM), at α=0.05. Charts were drawn with Excel software.

## Results

### Histology of the uterus

**Fig.1:**
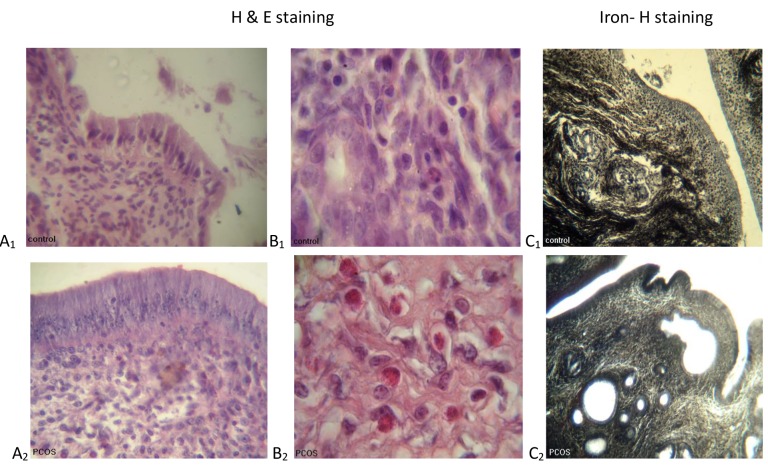
Histological sections of the uterus from control and estradiol valerate (EV)-treated polycystic ovary syndrome (PCOS) rats following hema- toxylin and eosin (H&E) and iron hematoxylin (iron-H) staining. A_1_ , A_2_. The uterine epithelium (×400), B_1_ , B_2_ . Eosinophil cells in the endometrial stroma (×1000), and C_1_ , C_2_ . The endometrial glands (×100).

**Fig.2:**
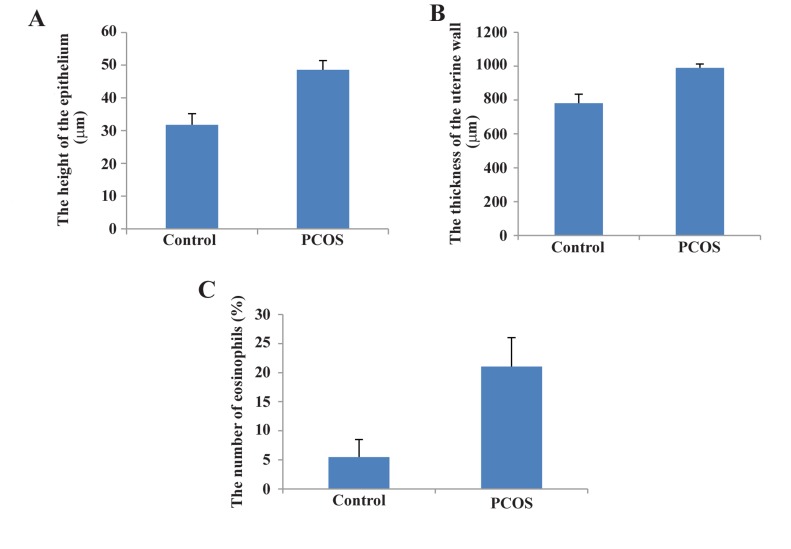
Statistical comparison between control and estradiol valerate (EV)-treated polycystic ovary syndrome (PCOS) rats. A. The height of the uter- ine epithelium (P<0.05), B. The thickness of the uterine wall (P<0.01), and C. The number of eosinophil cells in the endometrial stroma (P<0.05).

Microscopic study of the uterine tissue in the group treated by EV (PCOS) showed an increase in luminal epithelium height, accumulation of endometrial glands and their luminal diameter, and also the number of eosinophils in the endometrial stroma (Fig .1). Statistical comparison among the groups also revealed that luminal epithelium height and the thickness of the uterine wall in the PCOS group increased significantly compared to the control group (Fig .2A, B, Table 1). In addition, the percentage of eosinophils significantly increased in the experimental group (Fig .2C, Table 1).

**Table 1:** The height of the epithelial cells, uterine wall thicknesses, and the numbers of eosinophil cells in uterine stroma in control and estradiol valerate (EV)-treated polycystic ovary syndrome (PCOS) rats


Group	Cell height (µm)	Wall thickness (µm)	Eosinophils (%)

Control	31.81 ± 3.38	781.11 ± 53.59	5.49 ± 3.01
PCOS	48.57 ± 2.81*	989.96 ± 22.07†	21.06 ± 4.97*


Values are mean ± SE. *; P<0.05 and †; P<0.01.

Researchers have reported that eosinophilic infiltration may be under the control of different hormones in rats. Eosinophilic infiltration is dependent upon the continued presence of elevated levels of estrogen in the blood and 17β-estradiol stimulates eosinophilic invasion (32). Therefore, in the present study, we have documented changes in the numbers of eosinophils after the injection of EV as a hormonal mechanism.

### Proliferation

**Fig.3:**
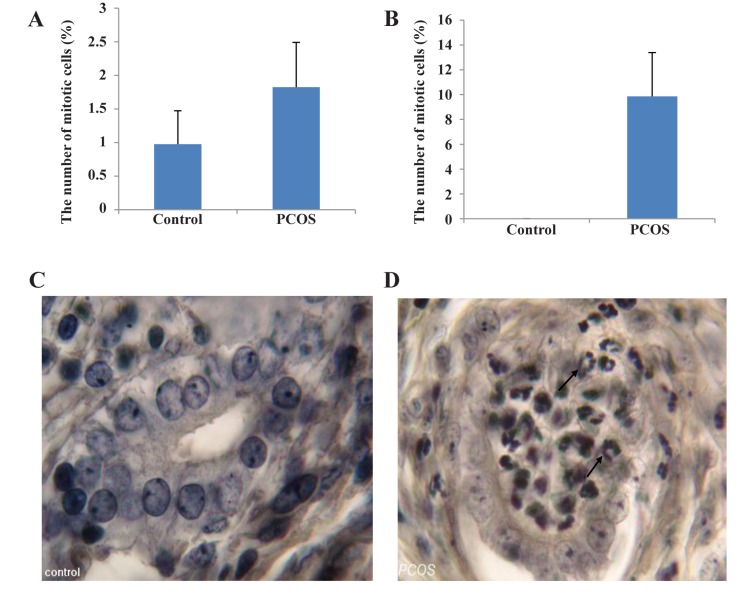
Up-Statistical comparison between control and estradiol valerate (EV)-treated polycystic ovary syndrome (PCOS) rats. A. The number of mitotic cells in the luminal epithelium (P<0.05), B. The number of mitotic cells in the glandular epithelium (P<0.001). Down-Histological sections of the uterus from control and EV-treated PCOS rats following iron hematoxylin (iron-H) staining, C, and D. The glandular epithelium (×1000). ↗; Mitotic cells

We examined and counted the epithelial cells in order to assess the frequency of mitotic cells in uterine luminal and glandular epithelia among the samples stained with iron-H. The numbers of mitotic cells were compared to the total numbers of epithelial cells. We assessed a total number of 2164 cells in the uterine luminal and 2195 cells in the glandular epithelia. A comparison between the groups revealed that the PCOS group had a nonsignificant increase in percentage of mitotic epithelial cells in the luminal region (Fig .3A, Table 2). On the other hand, the percentage of mitotic cells increased significantly in its glandular counterpart (Fig .3B, Table 2). Animals that received EV had remarkably more mitotic cells compared to animals in the control group (Fig .3C, D). 

**Table 2:** Percentage of mitotic cells in luminal and glandular epithelia in control and estradiol valerate (EV)-treated polycystic ovary syndrome (PCOS) rats


Group	Luminal epithelium (%)	Glandular epithelium (%)

Control	0.97 ± 0.49	0.00 ± 0.00
PCOS	1.82 ± 0.67	9.86 ± 3.53*


Values are mean ± SE. *; P<0.001.

It can be concluded that, as a sign of proliferation, the increase in numbers of mitotic cells leads to the development of a uterus with a thicker wall and dilated glands. This result can be considered as an overture for hyperplasia. 

## Discussion

The present study assessed the proliferative activity and histological changes in uterine tissue of an EV treated PCOS female rat model. Histological observations of uterine tissue sections showed a statistically reasonable increase in wall thickness of the uterus of EV treated (PCOS) rats in comparison with the control group. There was a significant rise in the average of the height of epithelial cells in PCOS rats compared to normal control rats. It has been shown that estrogen mediated stimulation of the uterus results in morphogenetic changes that include alterations in the type and morphology of luminal and glandular epithelia (24, 25). Similarly, the current study has proven that the stromal uterine glands of PCOS rats have larger luminal space and higher accumulation. *In vitro* studies of radiothymidine uptake by endometrium suggest that the maximal proliferation in uterine glands and stroma is chiefly associated with high concentrations of estradiol (33) and that ovarian steroids are among the most significant factors that affect both morphology and motility of the uterus (34). The results of this study have also supported the idea that noticeable changes in the epithelial surface, gland accumulation, and overall thickness of the uterus wall due to an abnormality at the level of ovarian steroids.

Based on the results, we observed a significantly higher eosinophil quantity in the endometrial stroma in the experimental group compared to the control rats. Experiments on the effect of hormonal perturbations on reproductive tissues suggested that the leukocyte invasion into these tissues have mainly occurred under hormone control. Eosinophil invasion is related to the continued presence of elevated blood estrogen levels as it is stimulated by estrogen (35). It has been reported that the immune system and inflammation are involved in the pathophysiological process of PCOS (36). Additionally, polymorphonuclear leukocyte infiltration may be relevant to an immunological process (37).

Results of the changes in proliferative activity in various regions of the uterus tissue showed a higher percentage of mitotic cells in luminal and glandular epithelia among rats of the experimental (PCOS) group compared to control rats. Estrogen has been well recognized as a strong factor which intensifies the proliferative activity of the uterus, with its major impact on uterine tissue (38, 39). The maximal proliferation in uterine glands and stroma occurs in the presence of high levels of estradiol (36). Studies have shown that the mitotic activity of estrogen in the endometrium of rodents is restricted to the luminal and gland neck epithelia (40, 41). In response to estrogen injection into ovariectomized mice, mitotic activity is first observed in the luminal, followed by the glandular region, while progesterone application can inhibit the mitotic response (42). Luminal epithelia have been suggested to undergo proliferation in the presence of 17β-estradiol (43, 44). In this study, we have shown that while mitotic activity was, to some extent, elevated in luminal epithelia in PCOS rats that received EV, this was not a statistically significant finding compared to the control group. We found that EV administration in PCOS rats had a surge in mitotic proliferation in the uterine glandular epithelia, which provided a probable explanation for the enlarged glands and thickened uteri wall. 

Increased estrogenic environment may favor mitogenic activity in the breast and/or other reproductive tissues (45, 46). Estrogens lead to a reduction in the duration of all the stages of the cell cycle and drive cells from the G0 to the G1-phase; this is followed by an increase in the number of cells in passing the G1-and S- phases, as well as the quantity of dividing cells (47-49). Hyperplasia is an early response to an abnormal stimulation in the cell proliferation process which leads to an increase in the numbers of cells. Hyperplasia can cause the organ size to increase. It has been suggested that the development of estrogen related morphogenetic changes in the uterus can be considered as an early step towards endometrial hyperplasia and cancer (50). The persistent stimulation of endometrial tissue by estrogen (mainly estrone) in PCOS patients without the progesterone-induced inhibition leads to uterine hyperplasia as a preliminary step to carcinoma (1). Cellular proliferation and apoptosis in the human endometrial tissue take place in a cyclic procedure as they are regulated by steroid hormone levels (20). In the normal endometrium, pro-apoptotic and anti-apoptotic factors are under fine regulation that leads to tissue homeostasis which can be disturbed by hormonal alterations (51, 52). The uterus response to estrogen requires changes in the expression of genes whose products regulate successive and functionally interlinked cellular processes. Researchers suggest that the earliest changes after 17β-ethinyl estradiol treatment occur in the expression genes whose products are involved in transcriptional regulation and signal transduction, followed by those involved in mRNA and protein synthesis, cell cycle regulation, DNA replication, cell proliferation and differentiation, apoptosis, tissue remodeling, and immunological responses (53).

## Conclusion

Administration of EV to induce an animal model of PCOS caused changes in epithelial height, uterus wall thickness, and the quantity of eosinophil cells. Additionally, PCOS rats showed considerably higher rates of proliferation in the glandular epithelium region of their uteri. Hence, it could be concluded that excessive estrogen content attributed to EV administration, caused an increase in the mitogenic activity of the uterus, which could be a prologue to endometrial hyperplasia and carcinoma.
